# Lovemarks and beyond: Examining the link between lovemarks and brand loyalty through customer advocacy in the automobile industry

**DOI:** 10.1371/journal.pone.0285193

**Published:** 2023-04-27

**Authors:** Nayab Javed, Syed Haider Khalil, Amir Ishaque, Syed Majid Khalil

**Affiliations:** 1 Institute of Business Studies and Leadership, Abdul Wali Khan University, Mardan, KP, Pakistan; 2 School of Management, Air University, Islamabad, Pakistan; 3 Management Sciences Department, Islamia College University, Peshawar, KP, Pakistan; University of Naples Federico II: Universita degli Studi di Napoli Federico II, ITALY

## Abstract

**Purpose:**

Despite the potential for businesses, our understanding of lovemark brands and their consequences is limited. Numerous psychological and brand-related consequences are linked to lovemarks, but the role of influential underlining mechanisms is not fully understood. Inspired by the norms of reciprocity theory, the current study investigates the underlining role of customer advocacy in the relationship between perceived lovemarks and brand loyalty of customers in the automobile industry.

**Methodology:**

By adopting the survey method, a sample of 478 was drawn from Pakistani automobile customers. Structural equation modelling was used for the analysis. We conceptualised lovemarks and brand loyalty as reflective higher-order constructs that we analysed through a two-stage disjoint analysis.

**Findings:**

Our results support the conceptualization of lovemarks and brand loyalty as higher-order constructs. The influence of lovemarks and customer advocacy on brand loyalty was statistically significant when we controlled for age, gender, and income. Our findings also reveal that customer advocacy, seen as a company’s positive interactions, mediates and play a key role in influencing the relationship between lovemarks and brand loyalty.

**Originality:**

This is among the first studies to examine the role of customer advocacy in the lovemarks-brand loyalty relationship. We examined these relationships in the automobile sector of Pakistan, which offers several theoretical and managerial implications for academia and practitioners. The implications are proposed and outlined in this study.

## 1. Introduction

Since the global car market has grown fiercely competitive, it has also affected Pakistan’s automobile industry. There are now more than a dozen new firms in the Pakistani automotive industry, which was formerly dominated by three Japanese automakers. Businesses have struggled to come up with innovative ways to serve their clients via informational exchanges that are rich in value, especially when we consider the significance of consumer empowerment in a highly connected society [1]. Customer advocacy has become a viable alternative strategy for enhancing firm performance outcomes by encouraging favourable customer reciprocal behaviours. One can argue that organisations succeed far more when they help buyers identify and get higher value in such market exchanges [2,3].

In today’s market, differentiating brands, products, and services on emotional value (such as relationships) is becoming highly important [4,5]. A prior study suggests that customer purchase decision is highly influenced by emotions, sentiments, and feelings than product/service characteristics and price worthiness [6]. In the circumstances involving purchases, emotions, and logic are interwoven. However, customers sometimes tend to utilise pseudo-logic to justify their emotional judgments and, in such situations, where emotions clash with reason, emotions take precedence in purchasing decisions [6]. Additionally, the fundamental distinction between emotion and reason is that the latter dictates the conclusions, while emotions shape human behaviour [4]. Building on this argument, one can argue that only emphasising satisfaction may be insufficient for building positive customer-brand relationships. The establishment and maintenance of strong brands via emotions and love for a brand are highly significant in building brand loyalty [4,7,8].

However, not all companies can build robust, long-lasting relations with their target audiences. Roberts [9] defined lovemarks as products, moments, and sentimental experiences that individuals fervently adore. Not all companies and their products turn into lovemark brands. The lovemark theory contends that such brands score well on the "love" and "respect" lovemark dimensions, which build consumer-brand interactions and therefore have a favourable impact on brand loyalty. Prevalent literature on lovemark suggests that lovemarks are not just an object (product brands) and can be conceptualised as relationship experiences [10]. Despite the importance of lovemarks, our understanding is limited about the interplay between lovemark brands and consumer behaviour.

A recent but significant development of the organisation-customer relationship lies in the understanding of customer advocacy. A recent study [11] suggests that advocacy can be seen from two perspectives: first, from the viewpoint of the customer (consumer advocacy), and second, as advocacy that is undertaken by the firms (customer advocacy). Consumer advocacy is the voluntary participation of customers in advancing positive word-of-mouth about the brands that can influence emotions, behaviours and purchase decisions [12]. On the other hand, Lawer and Knox [2] suggest that customer advocacy is a firm perspective and market orientation, which is driven by a desire to understand and respond to emerging consumer choices and their knowledge, and to gain customers’ involvement. Customer advocacy here refers to an organisation promoting the interests of customers [13].

There is a plethora of studies debating consumer loyalty, however, little is known about the antecedents and consequences of customer advocacy. To fill this gap, our research focuses on the critical examination of customer advocacy and its consequences. Customer advocacy in this sense can be seen as the act of organisations, working on behalf of their clients, advocating for them internally, and defending their best interests to enhance customer’s confidence and loyalty, and to secure positive repurchase intentions [3]. Customer advocacy also refers to representing and supporting customers’ interests with fairness and sincerity [2]. Attempting to promote brand loyalty, it is anticipated that organisations practising customer advocacy will enhance their relationships with customers, which can potentially build trust [14] and brand loyalty [15].

Brand loyalty is widely debated among researchers, yet there is little agreement on its causes and determinants. Particularly, little empirical evidence is available to explain how perceived lovemark brands interact with customer advocacy to influence brand loyalty in emerging countries [15,16]. The key role of underlying mechanisms, such as customer advocacy, is still evolving and remains an underexplored topic despite its significance for customer engagement and involvement [3]. According to academic research, context-specific factors such as industry type, culture, and country-specific traits may have an impact on consumers’ brand loyalty [17]. Despite the significance of lovemark brands, customer advocacy, and brand loyalty for automobile companies and their customers, little is known about how they interact and with what consequences.

Our study thus contributes to the knowledge about customer advocacy as a mediator of perceived lovemark brands and customer loyalty associations in the automobile sector of Pakistan. Drawing on the norms of reciprocity (NOR) that emphasise on the tit-for-tat behaviour and respond with benefits for benefits, the current study seeks an empirical validation of a mediation model where customer advocacy is conceptualised as a mediating variable (a reciprocating customer’s behaviour) between perceived lovemarks and brand loyalty (reciprocating action) in Pakistan’s automobile sector.

In terms of growth, the automobile sector in Pakistan has expanded exponentially, which is evident from a growth rate of 171% for the period 2014–2018 and still growing [18]. As per Census and Economic Information Center (CEIC) report, over 6.6 million registered vehicles were recorded by December 2021 [19]. Furthermore, Pakistan Automotive Manufacturers Association (PAMA) current report suggest a total of 226,433 cars were produced and 234,180 units of cars were sold in year 2021–22 [20]. Rasheed et al. [18] further argue that over 3.5 million people are working in the Pakistan automobile industry, which contributes about 3% of the GDP. In comparison to other Asian nations, Pakistan’s automobile market is relatively small but one of the leading fast-growing industries within Asian countries. Honda, Toyota, and Suzuki remained the three biggest brands in Pakistan’s car sector, however, Pakistan has seen a rapid increase in the popularity of vehicles made by South Korean, i.e., Kia Motors, and the Chinese manufacturer Changan in recent years [18]. With growing markets and industries come opportunities and challenges thus understanding the interplay of lovemarks customer advocacy and brand loyalty can provide some useful insights both for the producers and consumers of the automobile industry in Pakistan.

## 2. Literature review

### 2.1. Lovemark theory

According to Roberts [9], the two elements of a lovemark, i.e., love and respect, are the primary reasons behind unquestionable brand devotion. Respect not only depicts the performance of a brand, reputation and trust, per se but also symbolises the functional characteristics of a brand [4]. Brand love is the quality of a brand that are based on emotions that inspire customers for building an emotional relationship with the brand [9].

The concept of lovemarks strongly resonates with branding theory and with the significance of customer-brand associations. As organisations and their products become lovemarks, they focus on fostering and deepening their emotional connections with customers [6]. A product must have three essential components to be considered lovemarks. First, the mystery is portrayed through stirring tales from the past, myths, dreams, stories, and a lot of inspiration. Second, sensuality can be seen through the use of the five human senses. Finally, intimacy can be seen through dedication and empathy with the customer [9]. The concept of lovemark might explain why customers are more devoted to and committed to one brand than another.

#### 2.1.1. Brand love

Brand love can be seen as a deep emotional connection that a customer feels for a specific trade name [21]. Brand love is a personal, emotional, and devoted connection between a consumer and a brand that is distinguished by its reciprocity, purposiveness, and dynamic features [4], thus brand love is the affection a customer has for a company. Furthermore, brand love may only be experienced when both separation anxiety and deep affection are experienced simultaneously [22]. The foundation of the brand love notion is inspired by the “interpersonal love theory” when we attempted to examine the customer-brand relationships. Love may relate to an emotional state as well as a social connection. For instance, the love bond between two people is highlighted when people talk about lovers [23]. According to the triangular theory of love, there are three intricately connected aspects of interpersonal love i.e., intimacy, passion, and commitment. The “warm” characteristic of love, such as intimacy, can be seen as a sense of togetherness and bondedness. Passion on the other hand is the “hot” characteristic of love that promotes romance, sexual desire, and physical affection. Finally, Sternberg [23] concluded with the “cold” characteristic, such as commitment, which stands for a desire to love over a long term.

#### 2.1.2. Brand respect

Products must gain the respect of customers in addition to their affection to become lovemark brands. Based on the assessment of a brand’s performance, customers’ favourable view of a brand is expressed via brand respect [9]. Furthermore, prevalent literature suggests that a brand’s performance, trust, and reputation are said to be the three components that make up brand respect [9]. By delivering quality products, an organisation earns respect, which in turn builds trust and enhances its reputation. By lowering risk and raising performance expectations, the cognitive features of the brand image may promote trust [4] that can further improve customers’ confidence in the brand.

### 2.2. Customer advocacy versus consumer advocacy

Customer advocacy aims to create and maintain valuable connections by gaining the confidence of customers via open dialogue and collaborative efforts with them [2]. It has been argued that consumer activism and the perspective of the market can be seen as the origins of customer advocacy [16]. Consumer activism has an emphasis on customer benefits that aim for gaining trust, create lasting relationships, and generate positive word-of-mouth, by giving consumers desired knowledge and information to prevent negative experiences [17]. A prior study focused on the information-processing and the experience-based perspective of consumer behaviour [24]. Experience-based consumptions include imagination, feelings, and playfulness as processes that deliver opportunities for co-creation thus triggering superior customer learning. Information processing, on the other hand, is typically characterised by goal-oriented activities, for instance searching for alternative products during the purchase process [24]. A prior study suggests that consumer and customer advocacy may seem similar, yet they are conceptually different [25]. From a conceptual perspective, consumer advocacy reflects market/ product information sharing among consumers whereas customer advocacy is a construct used at the organisational level [25]. In other words, consumer advocacy is the self-initiated positive word-of-mouth customer behaviours that pass on to other customers whereas customer advocacy is an organisational strategy to identify emerging consumer choices via customer engagement and attempt to meet their demand anticipating that it will trigger positive reciprocal behaviours (i.e., word-of-mouth) and repurchase intentions.

Developing and maintaining customer advocacy continues to be a crucial strategy for businesses to gain a competitive edge, particularly when it comes to establishing client trust [26]. According to Urban [3], trust is highly significant for loyalty, which in turn will cause entry barriers. Advocates in this process are seen as dependable consultants who persuade current or future consumers to support the organisations throughout the purchasing process [27]. Yeh [26] contends that it is vital to spend on the improvement of customer relationship quality. Customers are eager to promote a company’s offerings when they get the degree of quality that they demand [28]. Customer advocacy is seen as highly significant as it can predict and enhance customer loyalty [29].

### 2.3. Brand loyalty

There is relatively little agreement among academics about the causes and effects of brand loyalty as region-specific factors including culture, industry, and other factors also affect consumer behaviour [17]. Customers’ brand commitment and attitude, which influences their behaviour toward the brand, can be termed as brand loyalty that helps customers to identify brands and their reputation [30]. Brand loyalty and the intention to make further purchases seem to be highly connected [15]. Quach et al. [17] argued that despite the competitor’s efforts and ability to encourage consumers towards switching brands, they will remain engaged with their brands and display repurchase intentions when brand loyalty is high.

Brand loyalty is a good predictor of future buying intention and creates favourable customer opinions that spread good word-of-mouth about the company [31]. Brand loyalty is a deliberate behaviour that is inspired by the consumer choice to create favourable customer outcomes, for instance, to remain loyal and to consider the brand in the repurchase process [32]. The drawback of focusing exclusively on repurchasing behaviour when analysing loyalty is that it leaves out key factors influencing customer loyalty [33]. It is believed that loyalty is a multifaceted concept that is influenced by both behaviour and psychological variables [15].

Oliver [34] presented a cascading model of loyalty that includes cognitive, affective, conative, and action loyalty. Cognitive loyalty is a common belief of a customer that a product is superior when compared to the competition [34]. A major drawback of such type of loyalty is that being developed in “cold” knowing, customers may switch loyalty when competitors offer a similar or superior product. Cognitive loyalty is inspired by how a customer thinks, affective loyalty, on the other hand, tends to be influenced by how customers feel [15]. Because of the affection one may have towards the brand, such customers are more loyal to the brand. The third level of loyalty i.e., conative loyalty, suggests that customers’ thinking and feeling generated by cognitive and affective loyalty encourages them to commit towards a repeat purchase. In this sense, conative loyalty can be seen as a motivation for taking action [35]. The action itself is the final stage of the loyalty cascade where customers make actual purchases or repurchases [15,34]. Action loyalty is thus the repurchase of customers that is influenced by the three theoretical phases of loyalty, i.e., cognitive (thinking), affective (feeling), and conative (motivation). Inspired by these arguments Quaye et al., [15] examined brand loyalty as a reflective higher-order construct, which was manifested by cognitive, affective, and conative loyalty as lower-order constructs. Conceptualising that the aforementioned three phases of loyalty drives repurchase intention (action loyalty), our study aligns with a prior study [15] that measured brand loyalty as a higher order construct that is seen through its three first-order dimensions i.e., cognitive, affective, and conative loyalty.

### 2.4. Norms of reciprocity theory

According to the NOR theory, individuals tend to reciprocate in kind for the favours they have received [36]. It may be interpreted as the expectation that individuals would reciprocate favourably with one another by exchanging advantages and reacting either indifferently or hostilely to harm. In many spheres of social life or various civilizations, the social principle of reciprocity often manifests itself in various forms. But each of these is unique from concepts like mutual goodwill and gratitude [36]. An underlying NOR is a potent tool on its own for fostering the cooperative behaviour necessary for self-sustaining social groups, limiting the harm done by the dishonest, and promoting the stability of social systems [37]. The exchange of benefits and good deeds is often confused with altruistic actions, but they are distinct [38]. Altruism can be seen as a voluntary act of involving in activities of proving benefits to the community/ society social without the expectation of return whereas reciprocal actions can be seen as reactions that follow others’ initial actions [38].

While the NOR theory specifically argues about the interaction between people where reciprocity takes place, we investigated the NOR theory by examining the company/product interaction with customers to examine the process of reciprocity. We thus argue that a positive perception of a company and its product (lovemark brands) along with customer-oriented actions and supportive interactions with customers (customer advocacy) is seen as a benefit and holds value for the customers. In return, customers attempt to pay this benefit by participating in reciprocal behaviours such as brand loyalty.

### 2.5. Hypothesis development

Perceived lovemarks are anticipated to be incredibly satisfying for consumers and can produce unquestionable brand loyalty [8,9]. The lovemark brand is anticipated to inspire customers to positively discuss their favourite brand with others and remain loyal, which in turn can provide an opportunity for the lovemark brands to charge premium prices.

Brand loyalty refers to the customer’s intent for remaining loyal to the brand, to recommend it to others, and to prefer the brand over competing brands [32]. Since customer recommendations are deliberate actions that have a positive impact on conative loyalty, El-Manstrly and Harrison [39] believe that it is a significant indication of conative loyalty. According to Shah and Khan [40], managers may choose business strategies that are distinctive and novel. To create effective business models, organisations must consider the viewpoints of their customers. Shah and Khan [40] further argued that organisations with creative skills exhibit stronger customer advocacy. During the interplay between loyalty and its antecedents, customer knowledge plays a vital role. Offering informed consumers intra-network promotion packages and marketing messages that highlights organisation’s innovativeness, will boost the likelihood of receiving favourable recommendations from consumers [15]. Furthermore, according to Urban [3], when organisations stand up for their customers, it can trigger reciprocating reactions from the customers, such as trust and repurchase intentions, thus promoting brand loyalty. We assert the following hypothesis:

H1: *Brand loyalty positively regresses on perceived lovemarks*.
H2: *Customer advocacy positively predicts brand loyalty*.

Lovemark brand improves the bond between customers and a brand, which consists of brand love and respect [4]. The process of building a consumer-brand connection will thus be driven by the emotional features of a brand that lead to the formation of brand love along with the functional aspects of a brand (i.e., brand respect). The suggested lovemarks metric, which in turn defines the nature of consumer-brand connections, is reflected in these two concepts. Roy [16] suggested that the foundation of customer advocacy revolves around the organisation’s capacity to meet its customer demands that in turn will induce reciprocating behaviours in the form of trust, love, and loyalty. We thus hypothesise:

H3: *Perceived lovemarks positively predict customer advocacy*.

Satisfaction, trust, and commitment are said to be indicators of enhanced relationship quality [41]. Brand loyalty enhances when customer perceptions are influenced by their prior positive experiences with the organisations [41]. Building on this stream of literature, it is expected that customer advocacy will mediate the link between perceived lovemarks and brand loyalty, therefore, we hypothesise:

H4: *Customer advocacy mediates the perceived lovemarks and brand loyalty association*.

**Fig 1:**
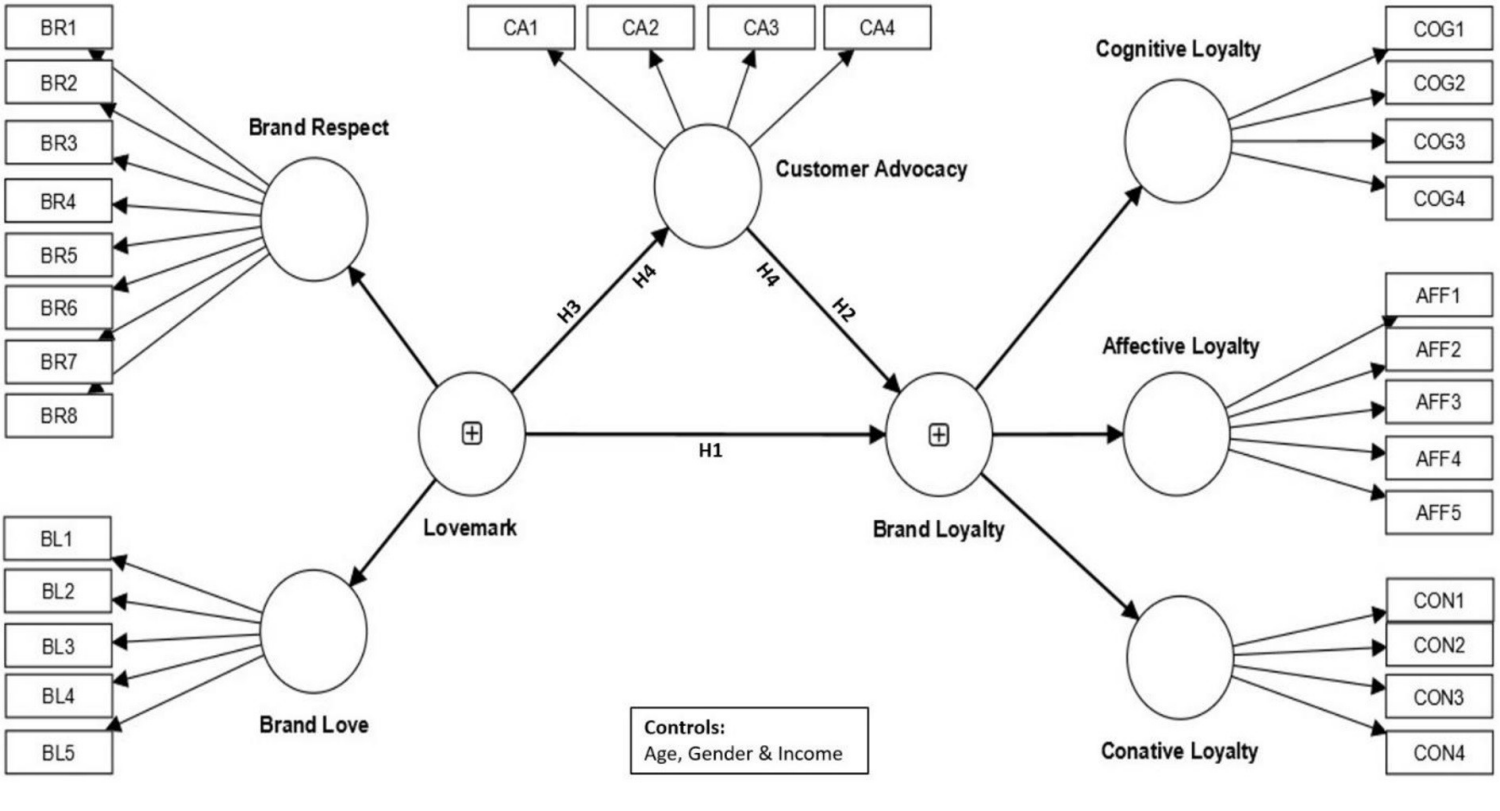
Conceptual model.

To summarise, we present Fig 1 which encapsulates the key variables, their manifest variables, and the hypothesized relationships. The model presents lovemarks as a reflective second-order construct manifested by brand respect and brand love as its first-order constructs. Brand loyalty is hypothesised as a reflective second-order construct that is manifested by cognitive, affective, and conative loyalty (lower-order dimensions).

## 3. Method

### 3.1. Ethics statement

The research ethics committee at the Institute of Business Studies and Leadership, Abdul Wali Khan University has approved the consent procedure of the current study. The data was gathered from adult respondents through online surveys as we have explained in the data collection section of our study. Participants were asked to read the consent form and provide written consent before participating in the study. Through the consent form, the participants were informed about the purpose of the study, and we confirmed that all responses will be used for academic purposes only. The current study also ensured the participants that their identities will be kept anonymous so that the data cannot be traced back to them in the published article.

### 3.2. Data collection and sample characteristics

An online survey of Pakistani automobile owners was conducted to collect the data for our study. The survey was hosted on Google Forms and sent out through email. The data was collected between January 15 to February 22, 2022. Identifying minimum sample size for multivariate analysis via structural equation modelling, a recent study recommends the use of power analysis [42]. We used a-priori sample size method for identifying sample size. By computing recommended information (effect size = 0.2, desired statistical power = 0.8, probability level = 0.05, unobserved variables = 7, and observed variable = 33) via power analysis procedure [43] the minimum sample size of 425 was identified for the current study. Considering that not all respondents would participate or may not complete the questionnaire (missing values), we assumed returned and useable questionnaires at 75%, thus a total of 567 (425/0.75) respondents were invited to take part in our study. We received 512 responses, and we used a total of 478 responses after the data-cleaning process to account for missing data and outliers. The respondents confirmed their involvement as active automobile owners. This ensured that only respondents who own a vehicle are included in our research study. A majority of respondents (78%) were men. The majority of respondents were between 30–40 years (36.6%) of age, which was followed by 19–30 years (26.4%). The majority of the respondents were earning between PKR 90,000 to PKR 150,000 (41.2%) followed by an income class of PKR 151,000 or more (31.4%). More than 34.5% of the respondents were from Pakistan’s Punjab Province, while 28.5% belonged to Khyber Pakhtunkhwa province.

### 3.3. Measures

All constructs were measured using the established scales from prior research studies. A recent study presented a third-order lovemark construct [4]. Giovanis & Athanasopoulou [4] conceptualised brand respect and brand love as second-order constructs of lovemarks (third order). Brand love was manifested by brand commitment, brand passion, and brand intimacy as first-order manifest variables. Similarly, brand respect was measured with its three first-order manifest variables (i.e., brand performance, brand reputation, and brand trust). In a recent study, all latent constructs were conceptualised as reflective constructs [4]. Since the reflective indicators are highly correlated and interchangeable, and removing items does not change the conceptual meaning of a construct [44], we used a shorter version of the lovemark construct to reduce model complexity. Aligning with Giovanis & Athanasopoulou [4] work, we measured lovemarks with their two sub-dimensions (i.e., brand love and brand respect). To measure brand respect, we used three items of brand performance, two items of brand reputation, and three items of brand trust. To measure brand love, we used two items of brand commitment, one item of brand intimacy, and two items of brand passion. All items were adapted from Giovanis & Athanasopoulou’s study [4].

We measured brand loyalty (i.e., reflective-reflective higher-order construct) with its three lower-order constructs (cognitive, affective, and conative loyalty) as conceptualised in a recent study [15]. We adapted a scale of 13 items used in Quaye’s et al. [15] study to assess brand loyalty sub-constructs. We measured cognitive loyalty with a four-item scale, affective loyalty with a five-item, and conative loyalty with a four-items scale.

We measured customer advocacy with a 4-items scale adapted from [16]. A five-point Likert scale was used for data collection. While considering brand loyalty as an outcome variable, a recent study controlled for age, education level, gender, and income [15]. While age, gender, and income provided some useful insights about the hypothesised relationships, respondents’ education level did not have a significant effect [15]. Building on these findings, we controlled for the effect of age, gender, and income to allow us for producing robust results.

## 4. Results

To determine the psychometric characteristics of each concept, we followed the recommended procedure for model analysis using SmartPLS 4 [45,46]. We used a two-stage disjoint analysis to perform measurement model analysis separately for first- and second-order latent variables.

### 4.1. Measurement model validation—lower level

**Fig 2:**
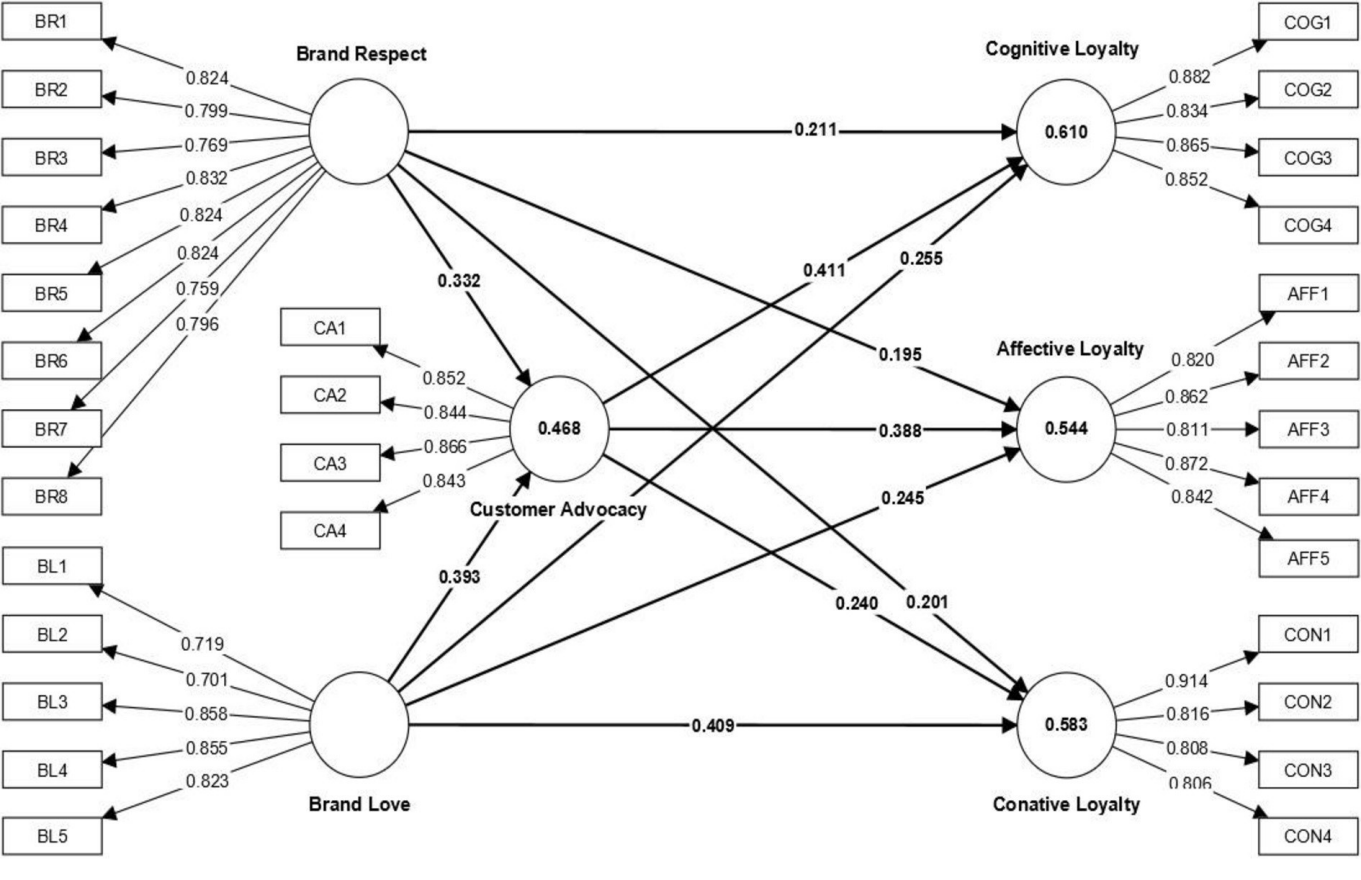
First-order estimates.

We started the analysis procedure by first examining the psychometric characteristics of the six first-order constructs using reflective indicators. Fig 2 presents a summarised version of the measurement model analysis.

The range of factor loading was between 0.701 to 0.914. The composite reliability was recorded between 0.894 to 0.936, which falls in the acceptable range i.e., between 0.70–0.95 [45]. Each latent factor’s AVE that depicts convergent validity, were between 0.630 to 0.737 (Table 1), which is higher than the cut-off value of 0.5 [47,48]. Since all first-order constructs were reflective, we used the recommended PLS consistent approach to provide more robust estimates [45]. Furthermore, the variance inflation factor (VIF) for all indicators were less than three suggesting no collinearity issues.

**Table 1:** First-order measurement model.

Lower-order Constructs	Indicators	Loadings	Cronbach’s alpha	(Rho_a)	(Rho_c)	AVE
Affective Loyalty	AFF1	0.820	0.924	0.924	0.924	0.708
	AFF2	0.862				
	AFF3	0.811				
	AFF4	0.872				
	AFF5	0.842				
Brand Love	BL1	0.719	0.894	0.9	0.894	0.63
	BL2	0.701				
	BL3	0.858				
	BL4	0.855				
	BL5	0.823				
Brand Respect	BR1	0.824	0.935	0.936	0.936	0.646
	BR2	0.799				
	BR3	0.769				
	BR4	0.832				
	BR5	0.824				
	BR6	0.824				
	BR7	0.759				
	BR8	0.796				
Customer Advocacy	CA1	0.852	0.913	0.913	0.913	0.725
	CA2	0.844				
	CA3	0.866				
	CA4	0.843				
Cognitive Loyalty	COG1	0.882	0.918	0.918	0.918	0.737
	COG2	0.834				
	COG3	0.865				
	COG4	0.852				
Conative Loyalty	CON1	0.914	0.904	0.906	0.903	0.701
	CON2	0.816				
	CON3	0.808				
	CON4	0.806				

Note: Rho_a and Rho_c = composite reliability, and AVE = average variance extracted.

Using the heterotrait-monotrait (HTMT) ratio, method of analysis for establishing discriminant validity, we followed the procedure explained in prior studies [49]. The HTMT is a more robust method for establishing discriminant validity as compared to the standard Fornell and Larcker [48] approach that only captures around 21% of discriminant validity whereas HTMT can capture 97–99% of discriminant validity [49]. Our study’s findings revealed the HTMT values to be less than the acceptable values (i.e., >0.85) for all first-order variables (Table 2).

**Table 2:** Discriminant validity statistics–HTMT.

Constructs	1	2	3	4	5	6
1. Affective Loyalty						
2. Brand Love	0.648					
3. Brand Respect	0.633	0.782				
4. Cognitive Loyalty	0.780	0.688	0.672			
5. Conative Loyalty	0.703	0.718	0.671	0.803		
6. Customer Advocacy	0.671	0.649	0.638	0.712	0.635	

### 4.2. Measurement model validation—higher level

The latent variable scores of brand respect, brand love, cognitive loyalty, conative loyalty, and affective loyalty were saved to the original data file to measure the reflective second order lovemarks variable.

Consistent with the process used for measuring first-order reflective variables, we achieved acceptable results for the perceived lovemarks variable using PLS consistent approach (Table 3). The factor loadings for all first-order constructs fall within the acceptable range thus establishing indicator reliability [45].

**Table 3:** Second-order measurement model.

Higher-order constructs	Lower-order constructs	Loadings	Cronbach’s alpha	(Rho_a)	(Rho_c)	AVE
Lovemarks	Brand Love	0.850	0.833	0.833	0.833	0.713
	Brand Respect	0.840				
Brand Loyalty	Affective Loyalty	0.826	0.875	0.876	0.875	0.700
	Cognitive Loyalty	0.864				
	Conative Loyalty	0.820				

The lovemarks and brand loyalty constructs also produced acceptable levels of CR and AVE values that are recommended for establishing reliability and convergent validity for latent variables [45]. Furthermore, we also check for the discriminant validity of higher-order constructs. The HTMT value of 0.770 between customer advocacy and brand loyalty and the HTMT value of 0.731 between lovemarks and customer advocacy meets the conservative cut-off value of <0.85. The HTMT value between lovemarks and brand loyalty (0.873) was higher than the conservative threshold value but less than the liberal cut-off value of 0.9 [45], which confirms that higher-order constructs were discriminant against each other.

### 4.3. Common method variance

We collected data from a single source through a survey, therefore common method variance (CMV) might be problematic [50]. To lower the probability of CMV, procedural and statistical remedies were used. To measure the variables, published and verified scales were used as a procedural remedy. Second, a cover letter, which ensured the privacy and confidentiality of the respondent’s personal information, was sent with the questionnaire. As a statistical solution for removing CMV, we used Kock’s [51] guidelines that are recommended for PLS-SEM. As evident from our analysis, the dataset lacks CMV since the latent variable VIF<3.3 [51]. From our CMV analysis, the results imply that the current dataset is free from CMV issues.

### 4.4. Hypothesis testing

**Fig 3:**
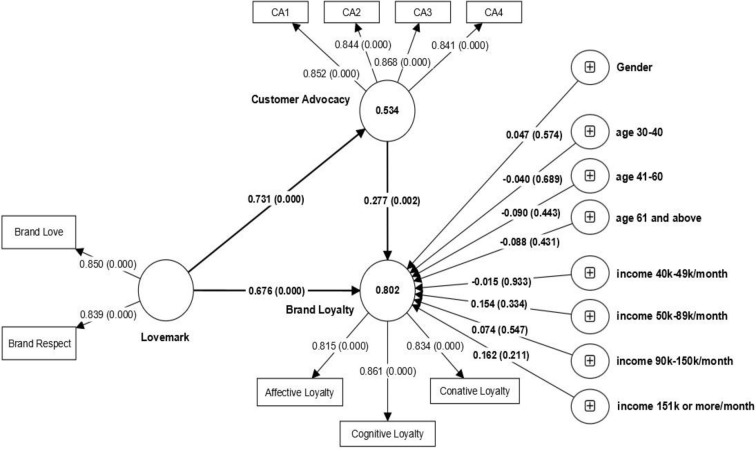
Structural model analysis.

The structural model analysis (Fig 3) was conducted once the measurement model was justified [52]. Bootstrap t-values were used to examine the significance of each relationship using ten thousand subsamples [53]. We then analysed the mediation effect of customer advocacy on the link between perceived lovemarks and brand loyalty.

The diagnostic metrics of the model (see Table 4) were deemed to be generally satisfactory [45] as R^2^ demonstrates a variance explained for customer advocacy and brand loyalty were 53.4% and 80.2% respectively, thus displaying medium to large explanatory power. The influence of lovemarks and customer advocacy on brand loyalty also has a moderate to large effect size (f^2^). Finally, we checked for predictive power using PLS Predict available in SmartPLS. The results from PLS Predict depict a medium to the strong predictive power of our model.

**Table 4:** Hypothesis testing results.

Hypothesis	Estimate	Std. Dev	T statistics	P values	f2	2.5%	97.5%
H1: Lovemark -> Brand Loyalty	0.676	0.079	8.559*	0.000	1.033	0.524	0.830
H2: Customer Advocacy -> Brand Loyalty	0.277	0.088	3.157*	0.002	0.180	0.104	0.444
H3: Lovemark -> Customer Advocacy	0.731	0.039	18.890*	0.000	1.146	0.649	0.801
Gender -> Brand Loyalty†	0.047	0.083	0.562	0.574	0.002	-0.116	0.209
age 30–40 -> Brand Loyalty†	-0.040	0.101	0.400	0.689	0.001	-0.247	0.149
age 41–60 -> Brand Loyalty†	-0.090	0.118	0.768	0.443	0.003	-0.324	0.133
age 61 and above -> Brand Loyalty†	-0.088	0.112	0.787	0.431	0.002	-0.308	0.130
income 151k or more/month -> Brand Loyalty†	0.162	0.129	1.251	0.211	0.008	-0.089	0.412
income 40k-49k/month -> Brand Loyalty†	-0.015	0.175	0.084	0.933	0.000	-0.357	0.334
income 50k-89k/month -> Brand Loyalty†	0.154	0.159	0.966	0.334	0.004	-0.148	0.474
income 90k-150k/month -> Brand Loyalty†	0.074	0.123	0.602	0.547	0.002	-0.159	0.322

†non-hypothesised relationships,

*p<0.01 (two-tailed).

The hypotheses test results presented in Table 4 show a positive direct effect of perceived lovemarks on brand loyalty (β = 0.521, t = 10.932, p<0.01) thus lending support to H1. The results show that customer advocacy (β = 0.358, t = 6.249, p<0.01) had positive direct effects on brand loyalty that we hypothesised (H2) in this study. Also, H3 came out statistically significant where we hypothesised that customer advocacy is positively predicted by perceived lovemark brands (β = 0.637, t = 17.86, p<0.01). During hypothesis testing, we considered the influence of age, Income, and gender by controlling its effects. Being categorical variables, we used the category of “below 30 years” for age and the category of “less than 40,000” for income as reference categories to test the significance of age and income. As evident from Table 4, the effect of age, income, and gender was non-significant.

### 4.5. Mediation effects

The guidelines provided by Nitzl et al. [54] for testing mediation in PLS-SEM were adhered to for investigating customer advocacy’s mediating role in the link between perceived lovemarks and brand loyalty relationships. Our results revealed that customer advocacy mediates the relationship between perceived lovemarks and brand loyalty (specific indirect effect = 0.202, t = 3.226, p<0.01, CI95 [0.080, 0.325]), thus supporting hypothesis H4. The positive direct effect and specific indirect effect suggest that customer advocacy partially mediates the perceived lovemark brands and brand loyalty relationship.

## 5. Discussion

The current study investigates the significance of customer advocacy in the relationship between perceived brand love and brand loyalty, where the findings of our study have significance for the marketing of automobiles in the Pakistani context. Customer actions represent our collective sense of being where advocacy behaviour becomes highly significant in protecting the welfare of the customers. Pakistanis place a significant emphasis on collectivism and cultural embeddedness that emphasise deriving life’s meaning from one’s social interactions with others, maintaining in-group cohesion and traditional order [55,56]. These characteristics are inheritably in contrast with Western civilizations, which place a strong emphasis on fostering individuality and autonomy [57]. The focus on lovemarks by Pakistan’s automobile executives in their efforts to engage in customer advocacy and to develop/ reinforce brand loyalty provide empirical evidence and aligns with the findings of prior studies [15], which were undertaken in low-context cultures, thus developing a consensus about the influence of the lovemark and customer advocacy on brand loyalty. In essence, lovemarks are very important to Pakistani automakers since they have a direct impact on a customer’s brand loyalty.

### 5.1. Theoretical implications

Our key theoretical contribution lies in the exploration of perceived lovemarks and brand loyalty association, and the mediating role of customer advocacy through the lens of NOR theory. Our findings highlight the importance of being a lovemark brand and customer advocacy in constructing brand loyalty. We argue that when customers perceive companies and their products as lovemark brands, along with the organisational effort to interact and listen to customer feedback (customer advocacy), it holds value for customers in such company-initiated social interactions. Being beneficial and valuable to the customers, lovemarks coupled with customer-oriented organisational effort (customer advocacy) trigger the norms of reciprocity among customers. In other words, customers value the effort and benefits offered by the firm are reciprocated by becoming loyal to the brands (i.e., brand loyalty) [58]. It became evident from our study that social ties can be enhanced by customer advocacy and consequently, brand loyalty will be reciprocated in such brand-customer exchanges.

Our findings also suggest that in the automobile sector, where customer advocacy is present, perceived lovemarks become quite important. In competitive markets, developing lovemark brands will allow an automotive business to create unique competencies that are hard to duplicate, thus giving the company a competitive edge [59]. Our results support Giovanis and Athanasopoulou’s [4] findings by demonstrating that brand loyalty, in the context of automobiles, is favourably predicted by perceived lovemarks. Our results also align with Alam et al. [29] findings by suggesting that brand loyalty is positively influenced when exposed to active customer advocacy. In addition, Roy [16] conceptualised customer advocacy as a sustainable strategy to account for consumers’ needs by promoting interactions between automobile manufacturers and customers that can trigger positive outcomes such as enhanced trust and loyalty. When customer interest becomes the employee’s top priority, it increases the quality of the relationship and, consequently, promotes brand loyalty [60].

### 5.2. Managerial implications

From a practical implication perspective, automobile manufacturers should emphasise customer advocacy, which further complements lovemarks in establishing loyalty among the customers of the automobile industry. This is important for automakers that may seek to enhance brand loyalty and gain additional resources. We argue that perceived lovemarks may also be employed as a strategy for improving one’s reputation to encourage favourable reciprocal behaviours, for instance enhancing favourable word-of-mouth about the organisations. While advocating for customers, organisations can place a strong emphasis on creating lovemark brands by giving customers fair, honest, and transparent advice. This makes it possible for businesses to gain the trust of their clients and motivates them for advocating their brand resulting in a reciprocal advantage (i.e., brand loyalty). Additionally, it instils a sense of pride and exclusivity in the consumers, igniting their enthusiasm for the lovemark brands and fostering a favourable perception of the companies, which strengthens brand loyalty and encourages desirable behaviours. Through cognitive, emotional, and conative loyalty, it establishes a favourable affinity for the firm. As a result, the automobile industry should acknowledge that lovemarks and customer advocacy may be able to help their brand co-creation strategies.

### 5.3. Limitations and directions for future studies

Some limitations can be attributed to our study. First, we used survey data that restricts statistical power and generalisability issues as to how broadly the results can be applied to the broader population. The scope of our study is further constrained since the information was mainly gathered from Pakistan’s large cities, which reflects a highly cosmopolitan view. Future research should broaden its geographic span to include new regions and countries. Subsequent research studies can expand into service sectors and other manufacturing industries. Lovemarks literature is dominated by examining its antecedents and limited outcome variables. It will be interesting to examine how the perception of lovemark brands interacts with other social behaviours and perceptions, for instance, brand image and brand equity. One could also think of exploring the influence of perceived lovemarks in an eco-friendly and sustainable environment and their association with the green practices of the firms.

## 6. Conclusion

The study’s main objective was to evaluate how perceived lovemarks influenced brand loyalty. To enhance the link between brand loyalty and customer advocacy, the current study analysed this relationship through the lens of customer advocacy as its key mediator. It thus attempts to present interconnectedness among key theoretical constructs that result in achieving superior brand loyalty and gaining much-needed competitive advantage in the ever-growing cutthroat competition. We used structural equation modelling as a mode of analysis to attain the set objectives of our study. The result of the current study confirms that perceived lovemarks can be seen as a key construct for building brand loyalty where the relationship is further enhanced in the presence of an effective customer advocacy strategy.

The results of our research add to the body of knowledge on brand loyalty and lovemarks. To the best of our knowledge, this is the first model concentrating on how brand loyalty in the automobile industry is influenced by perceived lovemarks and customer advocacy and validating their higher-order formations. By revealing the important role of customer advocacy as a mediator between perceived lovemarks and brand loyalty, this research expands our knowledge of lovemarks.

## Supporting information

S1 Data (XLSX)

S1 File Item scales and pls predict results. (DOCX)
