# Supplementary material for: Lovemarks and beyond: Examining the link between lovemarks and brand loyalty through customer advocacy in the automobile industry
Source: PLoS One. 2023 Apr 27;18(4):e0285193. doi: 10.1371/journal.pone.0285193 (PMC10138479; doi:10.1371/journal.pone.0285193)
Supplement: S1 File — (DOCX) [file pone.0285193.s002.docx]

**Constructs with scales and items**

| **Constructs** | **Code** | **Items** |
| --- | --- | --- |
| Cognitive loyalty | COG1 | My preferred car brand has more features than competing brands. |
| (4-items) | COG2 | The services offered by my preferred car brand are superior to rival car brands. |
|  | COG3 | I will always prefer my preferred car brand as they are more affordable than rival car/vehicles. |
|  | COG4 | My preferred car brand products and services are better than rival brands. |
| Affective loyalty | AFF1 | When I consider the available options, I like my preferred car brand. |
| (5-items) | AFF2 | The product and services of my preferred car brand are extra ordinary. |
|  | AFF3 | I enjoy using the products of my preferred car brand. |
|  | AFF4 | I am pleased with the preferred brand as they offer high quality products and services compared to other car brands. |
|  | AFF5 | It is always makes me happy to do business with my preferred car brand. |
| Conative loyalty | CON1 | I always promote my preferred car brand to others. |
| (4-items) | CON2 | It is highly likely that I will recommend my preferred car brand to others. |
|  | CON3 | During my discussion with friends and family, I always support my preferred car brand. |
|  | CON4 | I always advice my friends and family to consider my preferred car brand when purchasing a car/vehicle. |
| Customer advocacy | CA1 | I get honest advice from my preferred car brand, which helps me in choosing its product and services. |
| (4-items) | CA2 | I believe that my preferred car brand gives honest and full information to its customers about their products/services and competing products/services. |
|  | CA3 | My personal interests as a customer are highly valuable to my preferred car brand. |
|  | CA4 | My preferred car brand advocates for its customers. |
| Brand respect | BR1 | The product quality of my preferred car brand is very good. (Brand Performance) |
| (8-items) | BR2 | The product durability of my preferred car brand is very good. (Brand Performance) |
|  | BR3 | My preferred car brand performance is consistent. (Brand Performance) |
|  | BR4 | My preferred car brand is well-known. (Brand Reputation) |
|  | BR5 | My preferred car brand is highly reputable. (Brand Reputation) |
|  | BR6 | My preferred car brand always keeps their promises. (Brand Trust) |
|  | BR7 | My preferred car brand share honest information and do not make false claims. (Brand Trust) |
|  | BR8 | My preferred car brand is committed towards customer satisfaction. (Brand Trust) |
| Brand love | BL1 | I believe that I am highly committed towards my preferred car brand. (Brand Commitment) |
| (5-items) | BL2 | My preferred car brand is highly reliable. (Brand Commitment) |
|  | BL3 | I feel passionate towards my preferred car brand. (Brand Passion) |
|  | BL4 | My preferred car brand is captivating. (Brand Passion) |
|  | BL5 | I feel connected towards my preferred car brand. (Brand Intimacy) |

**PLS Predict Results**

| **Items** | **Q²predict** | **PLS-SEM RMSE** | | **PLS-SEM MAE** | | **LM_RMSE** | | **LM_MAE** | | **LM Less PLS (RMSE)** | |
| --- | --- | --- | --- | --- | --- | --- | --- | --- | --- | --- | --- |
| LAFF1 | 0.299 | 1.002 | 0.780 | | 1.016 | | 0.799 | | 0.014 | |  |
| AFF2 | 0.313 | 0.942 | 0.724 | | 0.954 | | 0.743 | | 0.012 | |  |
| AFF3 | 0.280 | 0.962 | 0.735 | | 0.978 | | 0.755 | | 0.016 | |  |
| AFF4 | 0.310 | 0.931 | 0.676 | | 0.945 | | 0.695 | | 0.014 | |  |
| AFF5 | 0.319 | 0.929 | 0.714 | | 0.944 | | 0.726 | | 0.015 | |  |
| COG1 | 0.368 | 1.059 | 0.790 | | 1.083 | | 0.813 | | 0.023 | |  |
| COG2 | 0.338 | 0.947 | 0.698 | | 0.959 | | 0.719 | | 0.012 | |  |
| COG3 | 0.359 | 1.043 | 0.757 | | 1.062 | | 0.780 | | 0.019 | |  |
| COG4 | 0.352 | 0.984 | 0.738 | | 0.999 | | 0.762 | | 0.014 | |  |
| CON1 | 0.445 | 0.976 | 0.735 | | 0.962 | | 0.718 | | -0.013 | |  |
| CON2 | 0.316 | 1.063 | 0.770 | | 1.085 | | 0.798 | | 0.022 | |  |
| CON3 | 0.333 | 1.078 | 0.843 | | 1.085 | | 0.862 | | 0.007 | |  |
| CON4 | 0.335 | 1.090 | 0.846 | | 1.110 | | 0.866 | | 0.020 | |  |
| CA1 | 0.313 | 1.081 | 0.809 | | 1.098 | | 0.828 | | 0.017 | |  |
| CA2 | 0.308 | 0.944 | 0.701 | | 0.961 | | 0.712 | | 0.016 | |  |
| CA3 | 0.333 | 1.044 | 0.774 | | 1.054 | | 0.786 | | 0.010 | |  |
| CA4 | 0.300 | 0.987 | 0.733 | | 1.000 | | 0.751 | | 0.013 | |  |

q2_predict > 0

All Items have LM>PLS (RMSE), except CON1 (Hair et al., 2021). RMSE was used for comparison as data was normally distributed.
